# Diffusion Correction in Fricke Hydrogel Dosimeters: A Deep Learning Approach with 2D and 3D Physics-Informed Neural Network Models

**DOI:** 10.3390/gels10090565

**Published:** 2024-08-30

**Authors:** Mattia Romeo, Grazia Cottone, Maria Cristina D’Oca, Antonio Bartolotta, Salvatore Gallo, Roberto Miraglia, Roberta Gerasia, Giuliana Milluzzo, Francesco Romano, Cesare Gagliardo, Fabio Di Martino, Francesco d’Errico, Maurizio Marrale

**Affiliations:** 1 Department of Physics and Chemistry “Emilio Segrè”, University of Palermo, Viale delle Scienze, Edificio 18, I-90128 Palermo, Italy; grazia.cottone@unipa.it (G.C.); mariacristina.doca@unipa.it (M.C.D.); antonio.bartolotta@unipa.it (A.B.); 2 Istituto Nazionale di Fisica Nucleare (INFN), Catania Division, Via Santa Sofia, 64, I-95123 Catania, Italy; giuliana.milluzzo@ct.infn.it (G.M.); francesco.romano@ct.infn.it (F.R.); 3 Department of Biological, Chemical and Pharmaceutical Sciences and Technologies, Viale delle Scienze, Edificio 16, I-90128 Palermo, Italy; 4 ATEN Center, University of Palermo, Viale delle Scienze, Edificio 18, I-90128 Palermo, Italy; 5 Department of Physics and Astronomy “Ettore Majorana”, University of Catania, Via Santa Sofia 64, I-95123 Catania, Italy; salvatore.gallo@unict.it; 6 IRCCS-ISMETT, Radiology Service, Via E. Tricomi, I-90127 Palermo, Italy; rmiraglia@ismett.edu (R.M.); rgerasia@ismett.edu (R.G.); 7 Department of Biomedicine, Neurosciences and Advanced Diagnostics, University of Palermo, Via del Vespro, 129, I-90127 Palermo, Italy; cesare.gagliardo@unipa.it; 8 Centro Pisano Ricerca e Implementazione Clinica Flash Radiotherapy (CPFR@CISUP), Presidio S. Chiara, ed. 18 Via Roma 67, I-56126 Pisa, Italy; f.dimartino@ao-pisa.toscana.it; 9 Fisica Sanitaria, Azienda Ospedaliero Universitaria Pisa AOUP, ed.18 Via Roma 67, I-56126 Pisa, Italy; 10 Istituto Nazionale di Fisica Nucleare (INFN), Pisa Division, Largo B. Pontecorvo 3, I-57127 Pisa, Italy; 11 School of Engineering, University of Pisa, Largo Lazzarino 1, I-56126 Pisa, Italy; francesco.derrico@unipi.it; 12 School of Medicine, Yale University, 333 Cedar St, New Haven, CT 06520, USA

**Keywords:** gel dosimetry, Fricke gels, diffusion, artificial intelligence, PINN

## Abstract

In this work an innovative approach was developed to address a significant challenge in the field of radiation dosimetry: the accurate measurement of spatial dose distributions using Fricke gel dosimeters. Hydrogels are widely used in radiation dosimetry due to their ability to simulate the tissue-equivalent properties of human tissue, making them ideal for measuring and mapping radiation dose distributions. Among the various gel dosimeters, Fricke gels exploit the radiation-induced oxidation of ferrous ions to ferric ions and are particularly notable due to their sensitivity. The concentration of ferric ions can be measured using various techniques, including magnetic resonance imaging (MRI) or spectrophotometry. While Fricke gels offer several advantages, a significant hurdle to their widespread application is the diffusion of ferric ions within the gel matrix. This phenomenon leads to a blurring of the dose distribution over time, compromising the accuracy of dose measurements. To mitigate the issue of ferric ion diffusion, researchers have explored various strategies such as the incorporation of additives or modification of the gel composition to either reduce the mobility of ferric ions or stabilize the gel matrix. The computational method proposed leverages the power of artificial intelligence, particularly deep learning, to mitigate the effects of ferric ion diffusion that can compromise measurement precision. By employing Physics Informed Neural Networks (PINNs), the method introduces a novel way to apply physical laws directly within the learning process, optimizing the network to adhere to the principles governing ion diffusion. This is particularly advantageous for solving the partial differential equations that describe the diffusion process in 2D and 3D. By inputting the spatial distribution of ferric ions at a given time, along with boundary conditions and the diffusion coefficient, the model can backtrack to accurately reconstruct the original ion distribution. This capability is crucial for enhancing the fidelity of 3D spatial dose measurements, ensuring that the data reflect the true dose distribution without the artifacts introduced by ion migration. Here, multidimensional models able to handle 2D and 3D data were developed and tested against dose distributions numerically evolved in time from 20 to 100 h. The results in terms of various metrics show a significant agreement in both 2D and 3D dose distributions. In particular, the mean square error of the prediction spans the range $1\times 10^{-6}$–$1\times 10^{-4}$, while the gamma analysis results in a 90–100% passing rate with 3%/2 mm, depending on the elapsed time, the type of distribution modeled and the dimensionality. This method could expand the applicability of Fricke gel dosimeters to a wider range of measurement tasks, from simple planar dose assessments to intricate volumetric analyses. The proposed technique holds great promise for overcoming the limitations imposed by ion diffusion in Fricke gel dosimeters.

## 1. Introduction

Radiotherapy (RT) is one of the main treatment modalities for neoplastic lesions. The goal of RT is to deliver a prescribed radiation dose (the energy deposited in matter by ionizing radiation per unit mass) to a tumor site while minimizing damage to healthy tissue [1]. The effectiveness of RT can be enhanced by conforming 3D-dose distributions to the shape of the tumor volume. Recent advancements in the field, including treatments such as Intensity Modulated Radiation Therapy (IMRT), Stereotactic Body Radiation Therapy (SBRT), and brachytherapy, demand accurate dosimetry methods [2]. For example, IMRT, combined with the use of multi-leaf collimators, can deliver radiation through multiple beams with varying shapes to establish a dose distribution that tightly conforms to the planned target volume while limiting radiation to critical organs. With advances in treatment delivery and patient immobilization techniques, greater tumor control can now be achieved. However, more complex treatments imply that any error in dose delivery can result in more serious complications. Therefore, accurate radiation dosimetry is essential to improve patient survival rates. Along with the increasing need for accurate 3D measurements of radiation dose, there is a compelling demand for dosimeters capable of adapting to shape changes of the organs or phantom under irradiation. In particular, dosimetric systems embedded in a hydrogel matrix ([3,4]) could offer the advantageous possibility of modifying their chemical structure to achieve the desired mechanical properties. Among these, the Fricke gel dosimeter (FG) may be a good candidate for introducing the appropriate chemical modifications to produce the expected physical-chemical properties. FGs are chemical dosimeters prepared by infusing a ferrous ammonium sulfate solution (the Fricke solution [5]) into a tissue-equivalent hydrogel matrix [6]. This system relies on the oxidation of ferrous ions in an aerated, acidic ferrous sulfate solution. The action of ionizing radiation on the solution causes water decomposition, and the resulting hydrogen atoms react with oxygen to produce the hydroperoxyl radical. A series of reactions then completes the conversion of ferrous ($Fe^{2+}$) ions to ferric ($Fe^{3+}$) ions. The final concentration of radiation-induced $Fe^{3+}$ ions is proportional to the absorbed dose up to saturation ([3,4]). As a result, the concentration of $Fe^{3+}$ is correlated with the absorbed dose through the following relationship:

(1) $$\left[Fe^{3+}\right]=\frac{10D\cdot G(Fe^{3+})\cdot \rho }{N_{A}e},$$

where *D* is the dose, $G(Fe^{3+})$ is the chemical yield of $Fe^{3+}$ (expressed as ions produced per 100 eV), ρ is the density in kg/L, $N_{A}$ is Avogadro’s constant and *e* is the number of joules per electronvolt. In what follows, we always refer to dose distributions to point to the ion concentration distributions.

In FG, the absorbed dose can be determined using Magnetic Resonance Imaging (MRI) ([7,8,9,10,11,12,13]), as the two iron ions differently influence the nuclear relaxation times of the surrounding protons, or with optical absorption spectroscopy due to a color change induced by redox indicators like Xylenol Orange (XO), Methylthymol Blue (MTB) or other chelating agents that can reduce the diffusion of $Fe^{3+}$ ions [14,15,16,17,18,19,20,21]. Preliminary applications of Fricke gels for various radiation therapy beams have been reported in literature [22,23,24,25,26,27,28,29,30,31,32,33,34,35,36,37,38,39,40].

One of the primary challenges in 3D Fricke dosimetry is the degradation of the three-dimensional dose distribution’s integrity over time, particularly following inhomogeneous irradiation. Despite the effectiveness of Fricke gels in dosimetry, one significant challenge is the diffusion of $Fe^{3+}$ ions after their formation. This behavior results in a blurred or inaccurate representation of the radiation dose distribution, particularly over extended periods of time. The primary concern with $Fe^{3+}$ ion diffusion is the reduction in spatial resolution of the dose distribution. As $Fe^{3+}$ ions diffuse, they cause the spread of the dose signal, leading to a less precise mapping of where radiation was absorbed. This blurring effect can be particularly problematic in high-gradient regions where accurate dose measurement is critical. $Fe^{3+}$ ion diffusion introduces temporal instability in the dose measurement. The longer the time between radiation exposure and readout, the greater the diffusion and the more significant the distortion in the dose distribution. This poses a challenge for accurate dosimetry, especially in scenarios where immediate readout is not feasible. The diffusion of $Fe^{3+}$ ions complicates the quantification of dose levels. As the $Fe^{3+}$ ions move away from their original site, they artificially lower the dose in high-dose regions and increase the apparent dose in low-dose regions, leading to inaccuracies in dose calculations.

The main approaches investigated to reduce the diffusion coefficient involve the incorporation of a chelating agent to diminish ion mobility and/or alteration of the gel structure to a tangled matrix which can impede ion movement [3,4]. A chelating agent usually adopted is the Xylenol orange which effectively reduces the diffusion coefficient by half but also notably reduces dose sensitivity. Regarding the gel matrix, comparatively, iron ions exhibit a higher diffusion coefficient in agar gel than in gelatine gel [41]. The lowest ion diffusion coefficients were observed in poly-vinyl-alcohol (PVA) Fricke gel [42,43], which is about half an order of magnitude lower than that of a standard agarose Fricke gel. The production of PVA gel involves repeated freezing and thawing cycles, enhancing its viscosity and cross-linking, thereby shortening the relaxation time [42,44]. PVA gel can also be synthesized chemically with agents like glutaraldehyde, which forms acetal bridges with the PVA through its aldehyde groups [3,44,45,46,47]. PVA-GTA Fricke gels exhibit an about double R1-dose sensitivity to that of standard agarose Fricke gels. Xylenol orange PVA-GTA Fricke gels show consistent dose sensitivity and ion diffusion coefficients across gelation temperatures ranging from 6 °C to 42 °C. In PVA-GTA Fricke gel dosimeters, reduced rates of auto-oxidation have been observed, with the caveat that the purity of PVA is of utmost importance [17,48,49,50,51]. Alternative chelating agents to xylenol orange, such as methylthymol blue (MTB) and 5-sulfosalicylic acid, were explored, with the latter serving as a substitute for sulfuric acid. Incorporating dimethyl sulfoxide, a free radical scavenger, into an MTB-PVA-GTA Fricke gel dosimeter has been shown to decrease the diffusion coefficient [16,18,19,20,21,52]. To further reduce the diffusion coefficient, other solutions employing anionic hydrogels, adding agents that increase viscosity, dispersing ferrous ions within liposomes, utilizing sorbent polymers, or modifying the hydrogel structure with complex-forming groups could be considered [4].

Although matrix gels can reduce diffusion processes, they do not eliminate them entirely. The dose distribution experiences a blurring effect caused by diffusion, which is influenced by the time elapsed between irradiation and measurement. In practical settings, machines like MRI or Optical Tomography scanners may not be readily accessible shortly after irradiation. This significantly restricts the use of Fricke gels in various fields, particularly in medical applications.

In a previous work [53], we applied artificial intelligence methods to correct the blurring effects in 1D dose distributions. In particular, we exploited the well-known Physics Informed Neural Network (PINN) method [54,55,56,57,58,59,60,61,62,63,64,65] to solve the backward time diffusion problem. The way a PINN model works is by exploiting loss functions derived from physics laws to optimize Neural Network (NN) parameters. Many relevant physical problems, such as ion diffusion, are governed by Partial Differential Equations (PDE), which in turn, could be used to construct a loss function and to train a NN. As established by the universal approximation theorem [66], a NN is a universal approximator, therefore, it becomes itself the PDE solution. The greatest advantage is that the NN can be trained by probing the domain in the continuum, sampling different random points at each iteration (or epoch), rather than relying on a fixed grid of points. As a result, the model is capable of providing a solution at any point in the domain, greatly enhancing its predictive capability. Additionally, the NN’s fast gradient computations, leveraging the parallel processing power of GPUs, significantly reduce the computational time needed to achieve a highly accurate solution.

The driving force in the $Fe^{3+}$ ion diffusion process observed in Fricke gels is primarily governed by the concentration gradient of the ions within the gel matrix. As stated by Fick’s first law, the diffusion flux is proportional to the negative gradient of the concentration. By combining Fick’s first law and the mass conservation in the absence of any chemical reaction, it is easy to derive Fick’s second law, which fully describes the time dependence of the $Fe^{3+}$ ion diffusion in the matrix. Assuming the medium is homogeneous and isotropic it follows that Fick’s second law resembles the heat equation:

(2) $$\frac{\partial }{\partial t}C\left(x,y,z,t\right)=D\;\nabla^{2}C\left(x,y,x,t\right)$$

where *C* represents the concentration of $Fe^{3+}$ ions and *D* is the diffusion coefficient. Based on Equation (2) there is one attempt to predict the distribution of $Fe^{3+}$ ions at the time of irradiation, $t_{0}$, given the known distribution at the measurement time, $t_{f}$.

In this work, we present the results achieved by applying PINN models to solve the backward diffusion PDE in both two-dimensional (2D) and three-dimensional (3D) spaces. The NNs were tested on simulated data, i.e., modeled distributions numerically evolved forward in time for 10 h. Results show a good agreement between predictions and modeled `true’ initial distributions, highlighting the predicting capability and the robustness of the PINN methodology in different types of domains for multi-dimensional tests.

## 2. Results and Discussion

### 2.1. 2D PINN Model Predictions: Rectangular Distribution

**Figure 1:**
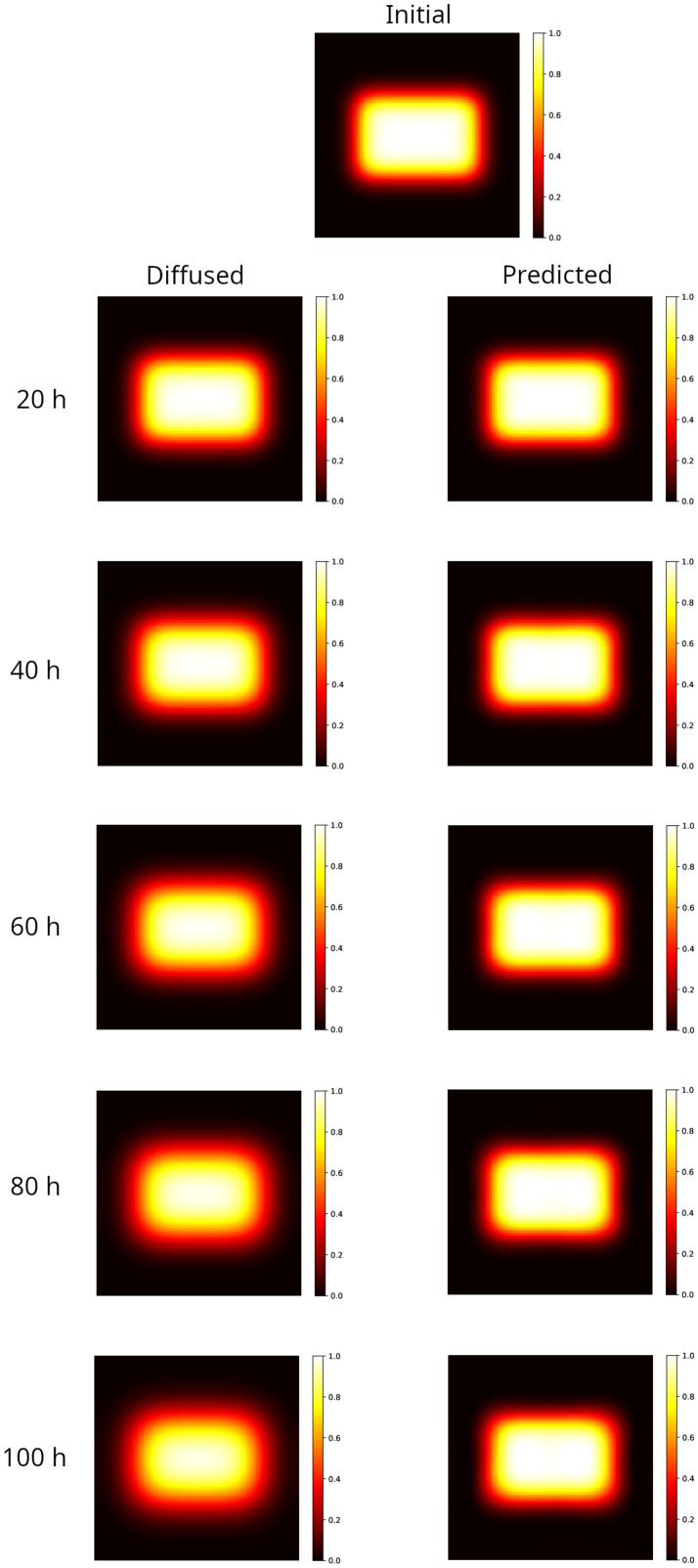
Modeling of a 2D sample partially irradiated using a uniform beam (rectangular spot area $40\times 60\;{\mathrm{mm}}^{2}$). Top image: initial distribution. **Left column**: distribution evolved forward in time, after 20–100 h. Note that as the time increases the hyper-intense area reduces, the edges become larger and the initial rectangular shape approaches an ellipse (100 h). **Right column**: reconstruction of the initial distribution, starting from the corresponding diffused on the left. The rectangular shape and the hyper-intense area are recovered, and the width of the edge matches the one in the initial distribution at all elapsed times.

Results are shown in Figure 1 for the rectangular distribution. The left panels show the distributions obtained by numerically integrating the Equation (2) at different times forward, starting from the initial distribution shown in the top panel of Figure 1. As time increases the hyper-intense area reduces, the edges become larger and the initial rectangular shape approaches an ellipse. This behavior, known as a blurring effect, is what compromises the accurate mapping of the radiation doses, limiting the reliability of the measurements performed within a few tens of hours after irradiation. The PINN method is able to reconstruct the initial rectangular shape starting from the corresponding diffused, reducing the edge width and correcting the hyper-intense area, as shown in the right panels’ maps in Figure 1.

To give a quantitative measure of the strength of the method, in Table 1 the errors of the prediction are reported with respect to the true initial condition, measured according to the different metrics described in Section 4.4. For the sake of comparison, the same metrics are applied to evaluate the distance between the initial condition and the final condition obtained by evolving the Equation (2) over different time intervals (from now on called diffused).

As can be observed, even at the longest simulated time ($t_{f}=100$ h, which is 1/6 of the characteristic time defined in Section 4), the true initial distribution is almost recovered by the PINN method. Indeed, the gamma analysis results in a 100% passing rate with 3%/2 mm, well above the rate set by the universal tolerance limits (95% at 3%/2 mm, [67]), whereas only the diffused distribution at 20 h obtains the same score. Furthermore, the MSE and 〈γ〉 are sizably lower in the reconstructed distribution with respect to the diffused one at all times.

### 2.2. 2D PINN Model Predictions: Circular Distribution

**Figure 2:**
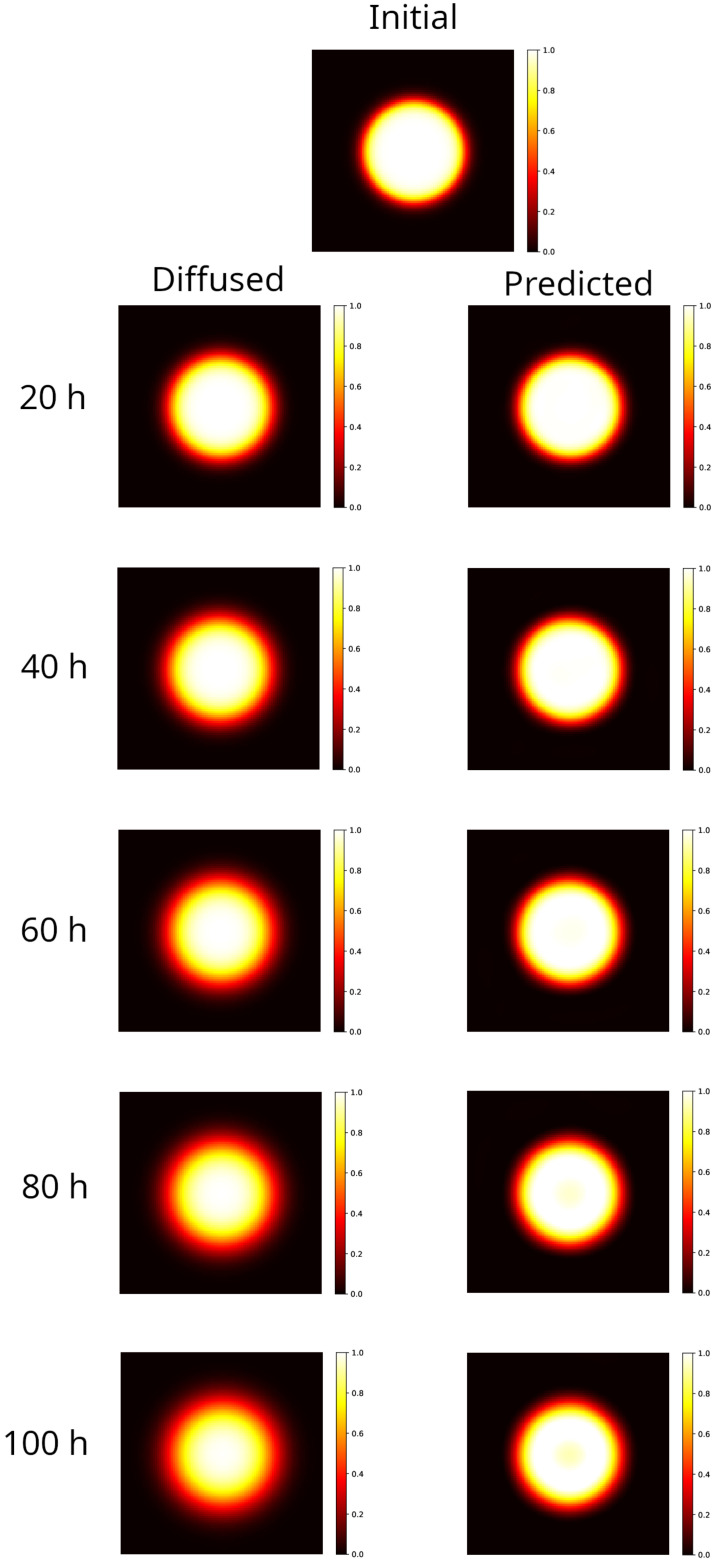
Modeling of a 2D sample partially irradiated using a uniform beam (circular spot radius 25 mm). **Top image**: initial distribution. **Left column**: distribution evolved forward in time, after 20–100 h. The blurring effect is apparent, as the hyper-intense area is sharp only at the center of the spot, while the edges blur, yet the circular symmetry remains intact. **Right column**: reconstruction of the initial distribution, starting from the corresponding diffused on the left. With respect to the rectangular case, the reconstruction becomes worse as time progresses. At 80–100 h, the model predictions exhibit a loss of signal at the center of the spot that could be, however, recovered by data post-processing.

As conducted for the rectangular distribution, in Figure 2 results are shown for the circular distribution evolved forward in time in the interval 20–100 h (left column panels), to be compared to the initial distribution (top panel). The blurring effect is noticeable: the hyper-intense region remains sharp only at the center of the spot, while the edges lack definition, though the circular symmetry is maintained. For the sake of comparison, the distributions reconstructed by the PINN method, starting from the corresponding diffused shown in the left panel, are also shown in the right panels of Figure 2.

The errors of the prediction with respect to the true initial condition, measured according to the different metrics described in Section 4.4, are reported in Table 2. As in the rectangular case, the same metrics are applied to evaluate the distance between the initial condition and the final (*diffused*) condition obtained by evolving the Equation (2) over different time intervals.

**Figure 3:**
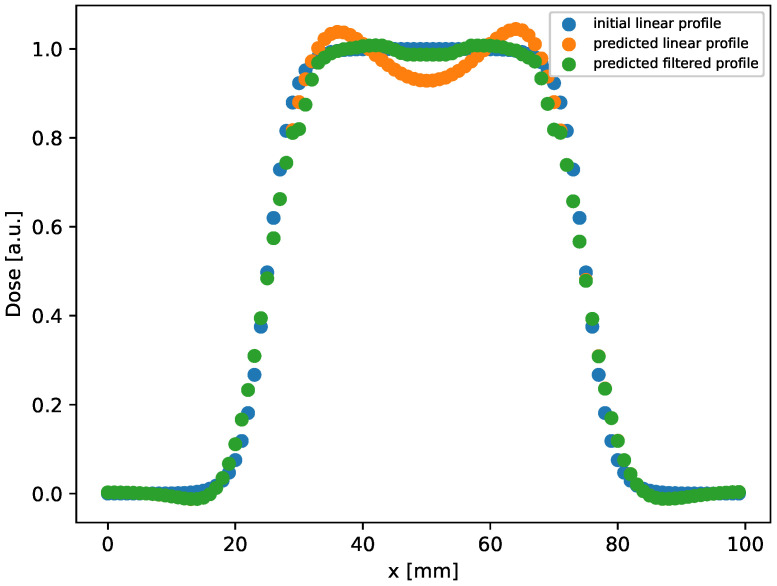
Linear profile obtained by selecting the central row (y=50 mm) in the distribution shown in Figure 2, right bottom panel, reconstructed after evolution at $t_{f}=100$ h. As can be observed, a good agreement between the initial and predicted profile is achieved by applying the filter.

**Figure 4:**
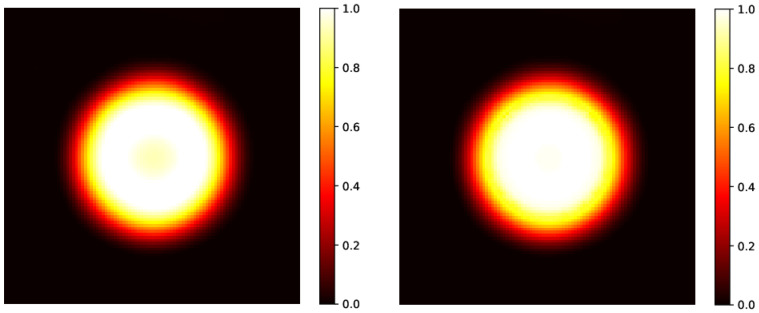
**Left panel**: predicted initial circular distribution without the application of a median 2D filter, already shown in Figure 2, right bottom panel, reconstructed after evolution at $t_{f}=100$ h. **Right panel**: predicted initial circular distribution after the application of the filter. As already shown in the linear profile in Figure 3, the initial hyper-intense area is almost recovered.

In this setup, the method provides results with an MSE in an order greater than the one in the rectangular case. In addition, we observe that the gamma index distribution sizably changes, as the population of points with γ index lower than 1 progressively decreases from 100% at $t_{f}=60$ h to 85% at the latest time ($t_{f}=100$ h). Indeed, as could be observed in Figure 2 at $t_{f}=100$ h (bottom right panel), and better evidenced in the linear profile shown in Figure 3 (orange circles), the reconstructed distribution exhibits a convex shape in its center, at variance with the true initial distribution (Figure 2, top panel and Figure 3, blue circles). This behavior, already observed in the 1D case [53], is probably due to the use of the hyperbolic tangent as activation function in the NN. It can be corrected by applying a selective median 2D filter. The result of this post-processing is shown in Figure 3, green circles, and in Figure 4 where the 2D map is shown and compared with the reconstruction without the filter. The %γ<1 increases to 97% at 3%/2 mm, to be compared with the value 85% with no filters.

### 2.3. 3D PINN Model Predictions: A DDP Model Distribution

**Figure 5:**
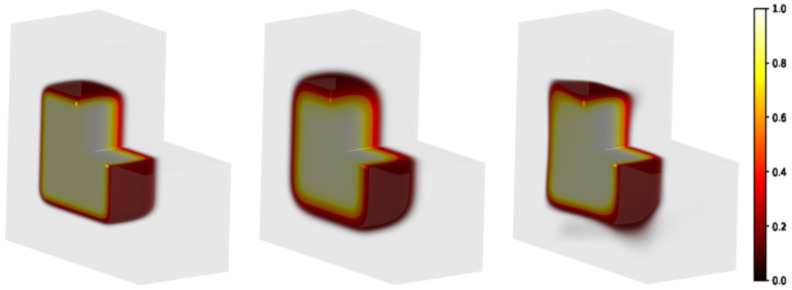
Modeling of a 3D sample partially irradiated using a uniform beam (the radiation hits the plan on the left in the figure). Square-spot area: $50\times 50\;{\mathrm{mm}}^{2}$. Matrix gel in light grey volume: $100\times 100\times 50\;{\mathrm{mm}}^{3}$. **Left panel**: initial distribution; **central panel**: distribution evolved forward in time, $t_{f}=40$ h; **right panel**: predicted initial distribution.

**Figure 6:**
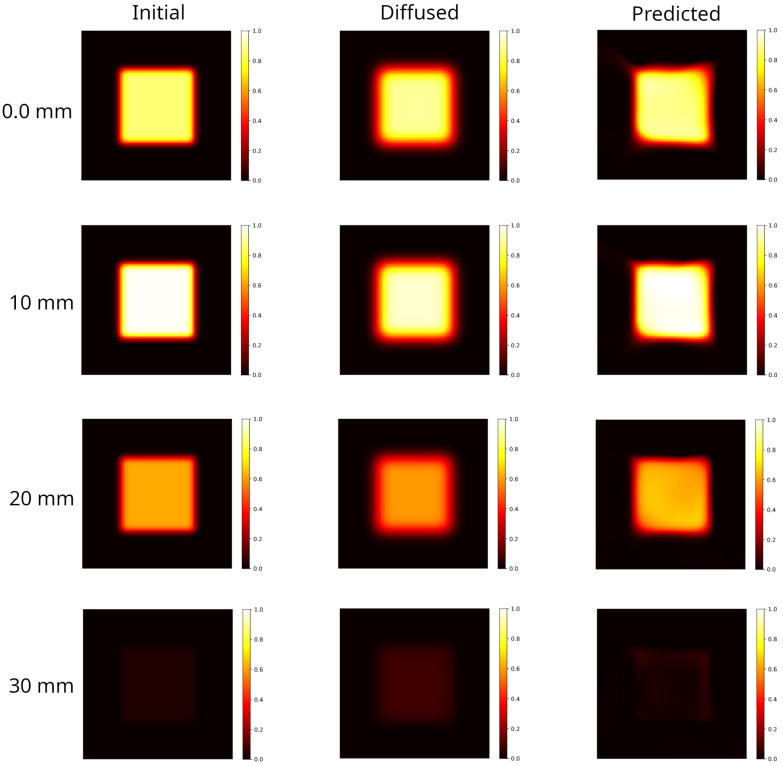
Selected axial slices of the 3D distributions shown in Figure 5. **Left column**: initial distribution. With reference to the DDP shown in Figure 9, the slices selected correspond to the input dose (z=0.0mm); the dose at the maximum value (z≈10mm); the half-maximum dose (z≈20mm); the approximately vanishing dose (z≈30mm). **Central column**: distribution evolved forward in time, at $t_{f}=40$ h. Blurring effects due to diffusion are evident. **Right column**: backward time model reconstruction of the initial condition, starting from the corresponding diffused distribution on its left. The PINN method is able to provide a better agreement with the DDP (see Figure 7). However, the model introduces artifacts at the edges which deforms the square shape.

The model ion distribution, shown in Figure 5 left panel, was generated in accordance with the in-depth dose profile at 7 MeV measured experimentally following the protocol fully detailed in Section 4.2. This distribution has evolved forward in time via the FTCS scheme up to $t_{f}=40$ h (Figure 5 central panel). The initial condition predicted by the PINN method is shown in Figure 5 right panel. For a clearer representation, in Figure 6 selected axial slices of the 3D distribution are shown, in the initial, diffused and predicted conditions. The slices have been selected referring to the DDP shown in Section 4.2 and in particular, correspond to: the input dose (z=0.0 mm); the dose peak (z≈10 mm); the half-maximum dose (z≈20 mm); the approximately vanishing dose (z≈30 mm).

Blurring effects due to diffusion lead to an enlargement of the irradiated region with a manifest reduction in the hyper-intense areas and a smoothed dose gradient. The reconstruction recovers the hyper-intense area and shape, the edges shrink although artifacts are present. Indeed, as can be observed in Figure 6 right panels, the model fails in reconstructing the corners where the dose gradients are larger.

**Figure 7:**
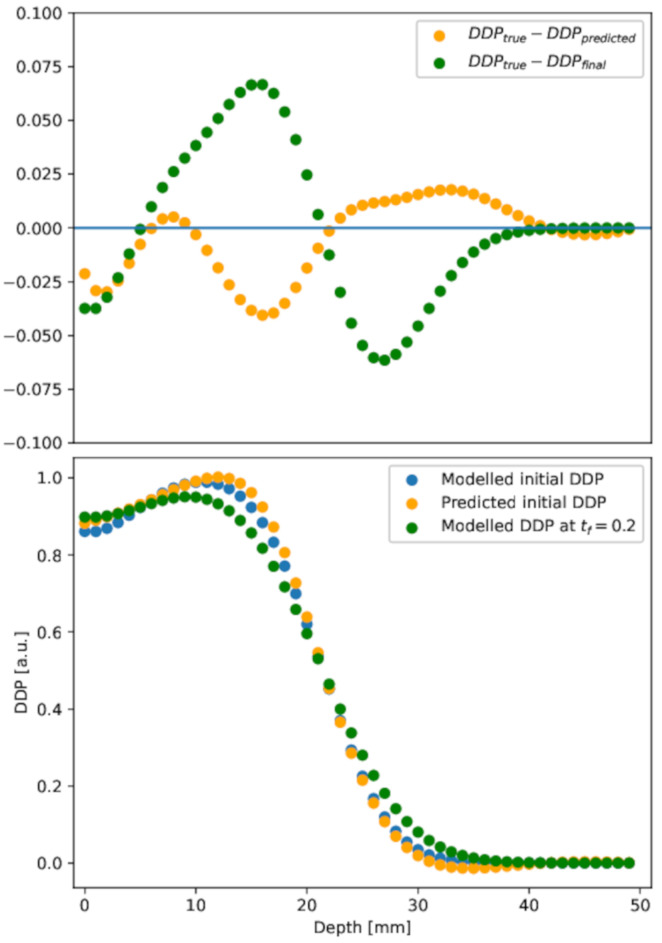
Depth dose profile. **Lower panel**: depth dose profile in the initial, diffused at $t_{f}=40$ h, and predicted initial conditions. **Upper panel**: difference between diffused or predicted initial depth dose profile and the initial one. The PINN result (orange points) is in good agreement with the initial distribution, at variance with the diffused (green points), in almost all depth ranges.

The DDP extracted from the reconstruction is shown in Figure 7, lower panel, orange circles. For the sake of comparison, the DDP extracted from the modeled initial condition (blue circles) and in the diffused distribution (green circles) are also shown. To assess the quality of the reconstruction method, the differences between profiles are highlighted in the upper panel of Figure 7.

**Figure 8:**
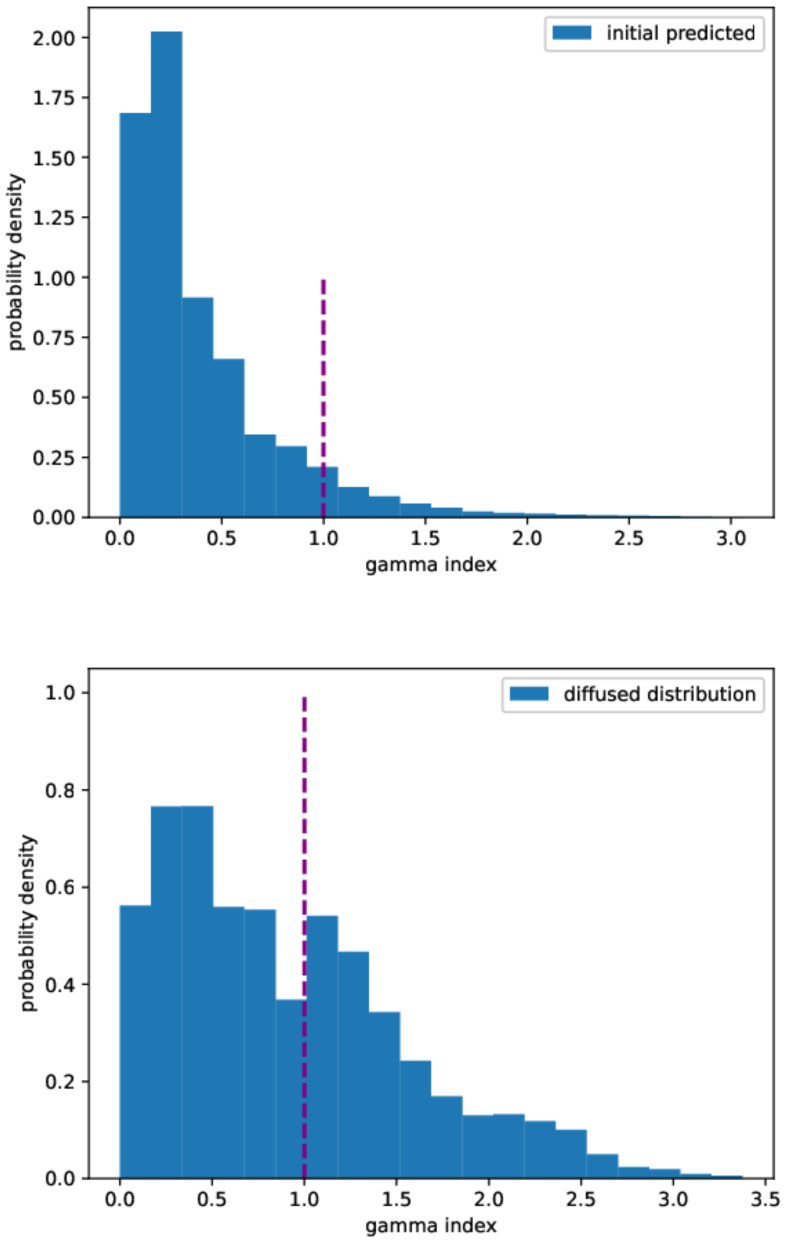
Gamma index distribution. **Upper panel**: predicted initial distribution. **Lower panel**: diffused distribution. Gamma index calculated at 3%/2 mm.

The errors of the prediction with respect to the true initial condition, measured according to the different metrics described in Section 4.4, are reported in Table 3. In particular, Figure 8 shows the gamma index distribution for both the initial predicted and the diffused distributions. The reconstruction method provides a better gamma index distribution, as the total number of points with a gamma index lower than 1 is about 92% to be compared with 60% in the diffused case. Although our passing rate is below the threshold set by the universal tolerance limits (95% at 3%/2 mm), it is above the value set by the universal action limits (90 % at 3%/2 mm) [67].

### 2.4. Discussion

Ferric ion diffusion in 3D dosimetric gels is a significant concern for their application in medical dosimetry, particularly in the accurate mapping of radiation doses within a three-dimensional space. Indeed, ferric ions ($Fe^{3+}$) produced after irradiation diffuse through the gel matrix over time. This diffusion can compromise the integrity of the dosimetric data and can lead to inaccuracies in the reconstruction of dose distributions (as shown by the γ index results on the diffused distribution) making it difficult to achieve accurate and reliable measurements, which are critical in radiation therapy. The primary issue is that as ferric ions migrate, they can cause an overestimation of the dose in low-dose regions and an underestimation in high-dose regions. Ferric ion diffusion can blur the dose gradients and result in a loss of spatial resolution in the dose distributions.

Several strategies to mitigate the problem of ferric ion diffusion in 3D gel dosimeters were developed and these primarily involve modifying the chemical composition of the gel matrix with the aim to either reduce the mobility of ferric ions and/or to stabilize their position within the gel. The incorporation of complexing agents, such as chelating compounds, into the gel formulation can effectively bind ferric ions, reducing their mobility. These agents form stable complexes with ferric ions, thereby limiting their diffusion through the gel matrix. Examples of complexing agents are xylenol orange, which is a pH indicator dye that forms a stable complex with ferric ions, inducing changes in the color of the dye that can also be used for optical density measurements in dosimetry [3,4]; sulfonated dyes, which can form strong ionic bonds with ferric ions, thus immobilizing them. Adding chelators (like xylenol orange) reduces ion mobility but sacrifices dose sensitivity [48]. Furthermore, the gel’s morphology could be altered to limit ion mobility inside the gel matrix. For example, polyvinyl alcohol (PVA) Fricke gels show small ion diffusion coefficients [42]). The gel viscosity is increased thanks to enhanced cross-linking obtained through cycles of freezing and thawing. Alternatively, PVA gel can be chemically cross-linked using agents like glutaraldehyde (GTA) [3,68]. Other chelating agents, such as methylthymol blue (MTB) and 5-sulfosalicylic acid, have also been investigated [16,18,69]. Recently, another research group assembled multiple emulsions coated with Fricke hydrogel and nanogel structures into hydrogel dosimeters, significantly reducing the diffusion coefficient of $Fe^{3+}$ [50,70,71]. Additionally, hydrophobic coatings proved to be highly effective in suppressing diffusion, a factor that was largely overlooked in previous studies of Fricke gel dosimeters.

In this work, we propose the use of Physics Informed Neural Networks (PINNs) to cope with the ion diffusion in Fricke gels. PINNs have emerged as a transformative approach in the field of computational science, particularly in solving complex differential equations that are inherent to physical processes. Recently, PINNs have been applied in solving the forward heat equation in 3D [72] and to solve 1D and 2D backward heat conduction problems [73]. In the context of 3D Fricke gel dosimetry, which is pivotal for the precise measurement of radiation doses in radiotherapy, PINNs could offer a significant advancement in addressing the challenge of ferric ion diffusion. The diffusion of ferric ions within the gel matrix is a critical factor that can affect the accuracy of dosimetric readings due to the resultant blurring of the dose distribution image over time.

A PINN can be trained to understand the underlying physics of ion diffusion by incorporating the governing physical laws into its learning process [54,55]. This is achieved by using the diffusion equation as a loss function during the training of the network. By doing so, the network not only learns from the data but also adheres to the physical principles, leading to a model that can generalize well to unseen scenarios. The potential of PINNs in this application lies in their ability to perform temporal reconstruction of the measured signal. Given the spatial distribution of $Fe^{3+}$ ions at a certain measurement time, along with the boundary conditions and the diffusion coefficient, a PINN can effectively reverse-engineer the initial condition of the system. This is particularly useful for correcting the diffusion effects post-measurement, thereby enhancing the fidelity of the dosimetric data.

This work extends a previous investigation in 1D that has demonstrated the feasibility of using PINNs for diffusion correction in Fricke hydrogel dosimeters [53]. Indeed, we proposed a computational method using deep learning to predict the initial condition of ion distribution in the gel, which showed promising results in both 1D simulated and experimental data.

Here, the same approach was able to recover the initial ion distributions in different model samples in 2D and 3D geometries. In the 2D case, different irradiation setups were exploited (rectangular and circular spot shapes). The PINN method was tested against distributions evolved in time by numerical simulations up to 100 h, in models of PVA-GTA B hydrogels ($D=0.25\;{\mathrm{mm}}^{2}/$h). The MSE of the prediction and the gamma analysis performed on the reconstructed initial distribution have shown significant improvement compared to the diffused distribution. In the case of a rectangular spot shape irradiation, PINNs provide initial distributions with 100%γ analysis passing rate (at 3%/2 mm), even at the longest time simulated (100 h). Similar results have been obtained with the circular spot-shape irradiation.

As for the 3D sample model, which is the most complex case study, although the MSE of the prediction is comparable to the one of the diffused distribution, the gamma analysis passing rate (92%) is above the value set by the universal action limit (90% at 3%/2 mm), demonstrating also in this case the capability of the reconstruction method.

However, the method here presented shows some limitations. The first is purely theoretical, as backward diffusion is an unwell-posed problem, i.e., it cannot be solved exactly. This introduces limitations on the time within which a reliable solution can be given. As it was shown, the goodness of the reconstruction becomes worse as the evolution time increases. Indeed the mean square error of the prediction increases by one order factor from $4.7\times 10^{-6}$ to $8.5\times 10^{-5}$ in the rectangular case, and from $2.7\times 10^{-5}$ to $6.6\times 10^{-4}$ in the case of circular distribution, as time increases from 20 to 100 h. In the latter case, also the gamma analysis shows a decrease in the passing rate from 100% to 85% (at 3%/2 mm) while keeping stable at 100% in the rectangular reconstructions.

With a view to using this approach in dose estimation, a study about the reconstruction ability as a function of the evolution time should be conducted taking into account how to model specific experimental factors such as type of gel, spot shape, space and time dependence of the diffusion coefficient, auto-oxidation effects, the presence of functional groups which could limit the ion diffusion, as well as the type of measurements (MRI or Absorbance). At present, our study just points out the capability and the potential of the method for diffusion correction; its application in dose estimation requires, however, fixing hyper-parameters and constraints in accordance with the experimental factors.

The method is not only sensible to the experimental set-up, but limitations can arise from the type of distribution investigated. For example, the linear profile plotted in Figure 3 for the model prediction in the case of the circular spot (orange curve) shows two bumps, not present in the true initial distribution. This behavior, observed in the previous 1D study too [53], could be associated with the hyper-parameters of the model and its own structure. Moreover, it is case-specific, in fact, it was not present in the reconstructed rectangular distribution. Furthermore, artifacts have been observed in the 3D reconstructed distributions at the edges where the dose gradients are high. Features such as network depth and structure, or the type of activation function should be explored for each case in order to find the best configuration. In our study a simple Fully Connected NN was used; other studies presented more sophisticated structures, such as Adaptive multi-scale NN [74], which may help in finding more accurate solutions. However, finding the best configuration anytime could lead to a non-affordable method due to the time required.

Last, another issue is related to the dimensionality: in the 3D case, the time required for the training is quite long, 9 h. In the scenario to analyze many measurements this time can represent a limitation. Several studies presented the use of adaptive activation functions which do not improve the results but allow the PINN to converge faster [75]. Other studies proposed the use of transfer learning to speed up training [76]. This approach, well known in the NN science, exploits the NN weights estimated in a previous training as a starting point for a new one. The simple idea is that PDE solutions can have several common features, so using pre-trained NN can skip the step where the model learns general properties and strictly moves to fine-tuning. In this way, the training can be shorter in time.

## 3. Conclusions

The diffusion of $Fe^{3+}$ ions in Fricke gels following irradiation is known to significantly limit their application across various fields. This is due to blurring effects that distort the dose distribution estimate, the extent of which depends on the time elapsed between irradiation and measurement. To address this issue, in this work, computational models are applied to reconstruct the initial dose distribution, which cannot be measured experimentally. Specifically, PINN models were developed to solve the backward diffusion equation. These models were tested using simulated ion concentrations in 2D and 3D model samples, mimicking concentrations at different times post-irradiation. These models were rigorously evaluated against time-evolved dose distributions ranging from 20 to 100 h. The evaluation metrics indicated a substantial concordance for dose distributions across both dimensions. Specifically, the mean square error in predictions varied between $1\times 10^{-6}$ and $1\times 10^{-4}$. Moreover, gamma analysis demonstrated a passing rate of 90–100% with a 3%/2 mm criterion, which varied based on the time elapsed, the specific distribution type being modeled (spot shape), and whether the data were in two or three dimensions.

Having bench cases from experimental measurements against which to compare the theoretical predictions would strengthen our computational protocol. At present, the model does not include some relevant factors present in an experimental setup. In the perspective of extracting reliable initial distributions from experimental data, some aspects could be explored such as (i) a modification in the diffusion equation to take into account source-loss terms modeling auto-oxidation phenomena and/or the presence of chelating agents; (ii) a modification in the structure of the NN itself; a modification in activation functions; transfer learning to reduce the necessary computational time.

While taking all this into account, the integration of PINNs into the analysis of 3D Fricke gel dosimetry still represents a significant leap forward in the quest for accurate and reliable dose measurements in radiotherapy. By leveraging the power of deep learning while being anchored to physical laws, PINNs hold the promise of overcoming one of the longstanding challenges in dosimetry. As research progresses, it is anticipated that such models will become an indispensable tool in the field, providing clinicians with the precision needed to deliver optimal treatment outcomes.

## 4. Materials and Methods

### 4.1. Sample Model Design

Simulations have been conducted by modeling simple setup irradiation. In particular, we modeled Fricke Hydrogel matrices, PVA-GTA B gels where $D=0.25\;{\mathrm{mm}}^{2}/$h [3], of size $100\times 100\;{\mathrm{mm}}^{2}$ in the 2D case and $100\times 100\times 50\;{\mathrm{mm}}^{3}$ in the 3D case, partially irradiated with uniform beams (25mm radius circular spot, $40\times 60\;{\mathrm{mm}}^{2}$ rectangular spot, $50\times 50\;{\mathrm{mm}}^{2}$ square spot). The 2D case is meant to mimic an experimental condition in which the axial dimension is sizably smaller than the other two dimensions, and the Depth Dose profile (DDP) could be assumed constant.

To construct an initial condition we start from a normalized distribution with the same shape of the beam spot, and we let it evolve forward in time for very few time steps. This provides a smoothed distribution to start with, better mimicking the real case.

**Figure 9:**
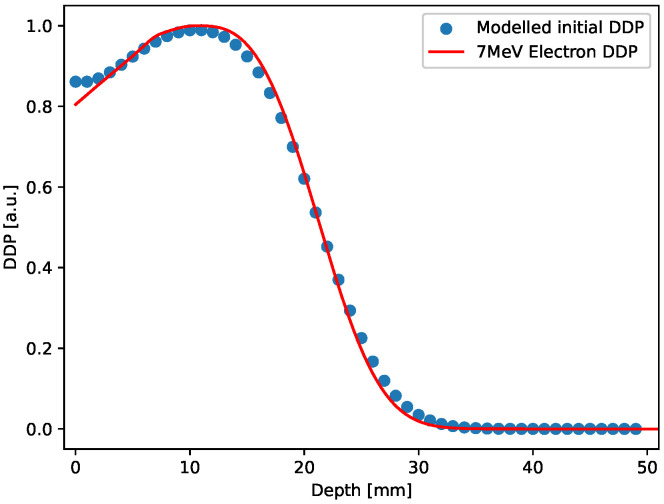
Red curve: Depth dose profile for a 7 MeV electron irradiation; blue circles: Depth dose profile extracted from the 3D initial distribution, after smoothing.

In the 3D case, the initial distribution has been constructed by stacking 50 2D uniform square distributions with values set according to the normalized Depth Dose profile (7 MeV electron irradiation) obtained for ultra-high dose rate electron beam produced using the electron FLASH linac (linear accelerator) currently installed at the Centro Pisano for FLASH Radiotherapy (CPFR) in Pisa (Italy) [77] and shown in Figure 9 (red curve).

This design only aims at providing an initial 3D distribution that mimics a realistic pattern generated by the radiation-matter interaction in gels. As in the 2D case, also the 3D sample generated was relaxed forward in time for a few time steps. The Depth dose profile extracted from the 3D sample after this small evolution is also shown in Figure 9, blue points, along with the experimental curve. The comparison shown in the figure points out that the modeled initial distribution actually reproduces a realistic case.

### 4.2. Numerical Integration of the Forward Diffusion Equation

Distributions are evolved by numerically solving the Equation (2) using *the Forward Time-Central Space (FTCS)* finite differences scheme on a discretized 2D or 3D space domain Ω [78]. Neumann boundary conditions were imposed,

(3) $$\frac{\partial }{\partial t}C\left(x,y,z,t\right)=0\;\;\;\;\;\;\;\;\;\;\forall t\;\;and\;\;x,y,z\in \partial \Omega \;$$

where ∂Ω is the domain boundary, which means that the amount of $Fe^{3+}$ ions in the initial distribution is conserved. This would be the case of low values of the auto-ionization constant. However, potential deviations could be incorporated into higher-order precision simulations, by modifying the second term of Equation (3) or introducing a source-loss term in Equation (2).

According to the stability analysis of parabolic PDE solutions obtained via the FTCS scheme [79], the maximum time step Δt allowed without the process becoming unstable is

(4) $$\Delta t=\frac{1}{2D\times (\frac{1}{\Delta x^{2}}+\frac{1}{\Delta y^{2}}+\frac{1}{\Delta z^{2}})}$$

Assuming the spatial grid resolution is the same in each dimension (Δx = Δy = Δz), the FTCS solution is stable when $\Delta t<\frac{1}{6D}$$\Delta x^{2}$ in 3D and $\Delta t<\frac{1}{4D}$$\Delta x^{2}$ in the 2D case. For numerical reasons, the calculations have been performed in arbitrary units, i.e., normalizing the spatial domain range to [0,1]; this gives in space 1a.u.=100mm. The diffusion coefficient was set to D=0.005a.u., to speed up calculations, and allow the upper limit of the time domain to be reached in a reasonable number of time steps. Given the gel matrix physical size is set to $100\times 100\;{\mathrm{mm}}^{2}$ and setting D to the value measured in realistic gels (PVA-GTA B), i.e., $D=0.25\;{\mathrm{mm}}^{2}/$h, the relation between the time in arbitrary units and physical units is as follows:

(5) 1timea.u.=200h

In our simulation we defined a grid of points with resolution $\Delta x=\Delta y=\Delta z=1\times 10^{-2}\;a.u.$, corresponding to 1 mm in each dimension. This results in a domain Ω=[0,1]×[0,1] in the 2D case and Ω=[0,1]×[0,1]×[0,0.5] in the 3D case, in a.u. To guarantee the stability of the solution the selected integration time step is $\Delta t=1\times 10^{-3}\;a.u.$ in 2D and $\Delta t=6.7\times 10^{-4}\;a.u.$ in the 3D case.

In order to have a bench case against which to test our reconstruction method, in the 2D case, for both rectangular and circular dose distributions, forward time simulations have been carried out to obtain the profile after 100, 200, 300, 400, 500 time steps, corresponding to a final time of 0.1, 0.2, 0.3, 0.4, and 0.5 a.u, i.e., to 20–100 h. The longest time scale simulated corresponds to a considerable fraction of the characteristic time required for the distribution to spread out over a distance equal to the radius of the circular spot, or equal to half of the average of the two rectangular dimensions of the spot (characteristic time 3.0 a.u. (600 h) for a characteristic distance of 0.25 a.u., in both cases). This characteristic time is estimated according to the time dependence of the mean square displacement in Brownian motion.

In the 3D case, the initial dose distribution has evolved forward in time over a 300 time steps time interval, corresponding to a final time of 0.2 a.u. (40 h), which corresponds to about 10% of the 3D characteristic time. As stated in the reference [53], as time progresses, information about the initial condition is lost leading to poorer reconstructions. In this respect, the characteristic time defines a threshold beyond which any reconstruction algorithm fails. This is due to the fact that, on average, in this time interval any particle has traveled a distance greater than the beam spot dimension.

### 4.3. PINN Model

Physics Informed Neural Network refers to a NN that incorporates principles of physics into its training. This approach utilizes a semi-supervised machine learning method, which means that the model’s training is data-driven and also incorporates additional information in the form of Physics equations.

Let us denote the position vector in a three-dimensional space as x. We call NN(x,t;θ) a generic NN with parameters θ, whose input is a point x in space at a certain time *t*. To force a PINN model NN(x,t;θ) in respecting a given equation, the parameters θ have to be optimized making use of an *ad-hoc* built loss function. The most straightforward way to build a loss function from a Physics equation is by calculating the MSE of the residuals over a set of testing points. So, starting from the diffusion equation (Equation (2)) and given a set of *N* points $\left(x_{i},t_{i}\right)\in \Omega \times \left[0,t\right]=A$, the MSE of the residual is as follows:

(6) $$MSE_{res}=\frac{1}{N}\sum_{i}{\left(\frac{\partial }{\partial t}C\left(x_{i},t_{i};\theta \right)-\;D\nabla^{2}C\left(x_{i},t_{i};\theta \right)\right)}^{2}.$$

Since PINN models exploit the Universal Approximation Theorem [66] to approximate the solution of the equation, the desired loss function can be obtained by replacing the concentration C(x,t) with the NN(x,t;θ):

(7) $$L_{PDE}=\frac{1}{N}\sum_{i}{\left(\frac{\partial }{\partial t}NN\left(x_{i},t_{i};\theta \right)-\;D\nabla^{2}NN\left(x_{i},t_{i};\theta \right)\right)}^{2}$$

From the mathematical and physical point of view, a boundary value problem (BVP) cannot be solved by just Equation (2), boundary conditions are needed as well. By imposing the Neumann boundary condition (Equation (3)) we can build one more loss function:

(8) $$L_{BC}=\frac{1}{N^{\prime }}\sum_{k}{\left(\frac{\partial }{\partial t}NN\left(x_{k},t_{k};\theta \right)\right)}^{2},$$

calculated over the set of $N^{\prime }$ points $\left(x_{k},t_{k}\right)\in \partial \Omega \times \left[0,t\right]=B$.

Equations (7) and (8) represent the unsupervised part of the PINN model’s training. The data-driven learning is infused by imposing that the NN is equal to the experimental or simulated distribution f(x) at some time $t^{\prime }$. In this way, we obtain the latest loss function:

(9) $$L_{FC}=\frac{1}{N^{\prime \prime }}\sum_{j}{\left(f\left(x_{j}\right)-NN\left(x_{j},t^{\prime };\theta \right)\right)}^{2},$$

calculated over the set of $N^{\prime \prime }$ points $\left(x_{j},t_{j}\right)\in \Omega \times \left\{t^{\prime }\right\}=C$.

The backward integration can be implemented by a clever selection of the time domain, the interval $\left[0,t_{f}\right]$, and imposing $t^{\prime }=t_{f}$ and $f\left(x\right)=C\left(x,t_{f}\right)$, where $t_{f}$ represents the time displacement between irradiation and measurements. In this way all time coordinates lie before $t_{f}$ and the algorithm provides the backward solution.

The strength of this method resides in exploiting the domain definition to implement backward integration, which would otherwise necessitate transformations in the equation, such as time inversion. The latter approach cannot be applied to the diffusion equation, which is not invariant under this transformation. Changing the sign of the second term in Equation (2) would imply a negative diffusion coefficient, which is not physically realistic. Differently, using a PINN model means fitting the domain $\Omega \times [0,t_{f}]$ in the space of the solutions of the BVP. Since the backward diffusion equation is an unwell-posed problem, i.e., the uniqueness of the solution is not granted, and we obtain just one of the possible solutions.

The collective sum $L=L_{PDE}+L_{FC}+L_{BC}$ is utilized to construct the total loss function. This function is instrumental in optimizing the NN to resolve the inverse problem effectively:

(10) $$\left\{\begin{array}{cc} \frac{\partial }{\partial t}C\left(x,t\right)=D\nabla^{2}C\left(x,t\right) & \left(x,t\right)\in \Omega \times \left[0,t_{f}\right]\\ \frac{\partial }{\partial t}C\left(x,t\right)=0 & \left(x,t\right)\in \partial \Omega \times \left[0,t_{f}\right]\\ C(x,t)=f(x) & \left(x,t\right)\in \Omega \times \left\{t_{f}\right\}. \end{array}\right.$$

Typically, the concentration at final time $t_{f}$, expressed as $C(x,t_{f})$, is known at a limited set of points. To estimate values across the entire spatial domain, an interpolation method is employed. Specifically, the Scipy library’s interpolation tools ([80]) are applied. The NN training follows a structured sequence of steps:

- Sample *N* couples $(x_{i},t_{i})\in A$, $N^{\prime }$ couples in $(x_{j},t_{j})\in B$ and $N^{\prime \prime }$ couples in $(x_{k},t_{k})\in C$;
- calculate $NN(x_{i},t_{i})$, $NN(x_{j},t_{j})$ and $NN(x_{k},t_{k})$;
- calculate L;
- optimize the parameters of the network θ;
- repeat the above steps $N_{epochs}$ time.

**Figure 10:**
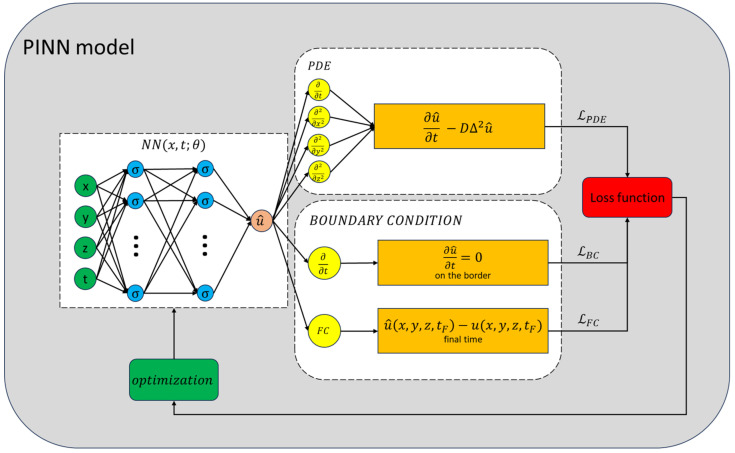
Scheme of the training process of the PINN model. The NN’s output is evaluated by the loss functions. The total loss function serves as an indicator of the model’s efficacy and guides the optimization algorithm in adjusting the parameters. This iterative process continues until the NN’s error is sufficiently minimized.

An illustrative diagram of the PINN model’s training methodology is depicted in Figure 10.

Model regularization is achieved by setting a predefined number of epochs and preserving the parameter configuration upon improved model performance. Training concludes when the loss function falls below a predetermined threshold ($1\times 10^{-5}$); otherwise, additional epochs are implemented.

The network we used is a Multi Layer Perceptron (MLP) with seven layers: an input layer, five dense hidden layers ($units=\left[10,20,200,20,10\right]$), and an output layer with one unit. The total number of the parameters of the network is 8691. We used the hyperbolic tangent as an activation function for all hidden layers, to ensure the derivability of the NN. As an optimizing algorithm, we used *Adam* ([81]).

In the 2D case, we trained the network for 100,000 epochs, sampling N=20,000, $N^{\prime }=20,000,$ N″ = 1000 points at each epoch, using the Python library Tensorflow [82] and its sub-library Keras [83]. The time required to train the network is about 1.5 h in the 2D case. In the 3D case parameters are as follows: 200,000 epochs, sampling N=100,000,$N^{\prime }=80,000,$ $N^{\prime \prime }=80,000$ points at each epoch. The time required to train the network is about 9.0 h.

### 4.4. Evaluation Criteria of the PINN Model Prediction

Once a model has been trained in agreement with the above protocol, it is possible to predict the initial distribution. In order to evaluate this approach it is necessary to compare the prediction with the ground truth, e.g., the true initial distribution. As it was conducted in the reference [53], an evaluation criterion is the Mean Squared Error (MSE), defined as:

(11) $$MSE=\frac{1}{N}\sum_{x_{i}}{\left(C\left(x_{i},t_{0}\right)-NN\left(x_{i},t_{0}\right)\right)}^{2}$$

where $C(x_{i},t_{0})$ and $NN(x_{i},t_{0})$ are the spatial concentration and the NN prediction at the initial time, and the sum is extended to all *N* points of the discrete domain. However, when the Region of Interest (ROI), e.g., the portion of the space mainly irradiated, is small compared with the size of the whole domain, MSE can be small even though prediction and ground truth are very different. This is due to the statistical effect of a large number of pixels/voxels which assume a value close to or equal to zero. Moreover, MSE is a global metric so we cannot use it to evaluate local features. For these reasons, we designed an evaluation criterion that includes gamma analysis (see Section 4.4.1) in addition to the MSE. Additional metrics (discrete and dosimetry) have been evaluated whose outcomes are reported in the supplementary materials. In this work, we reported results obtained by applying the MSE and gamma analysis to assess the reconstructed initial against the true initial distribution.

#### 4.4.1. Gamma Analysis

The gamma index (γ) is one of the most commonly used metrics in the radiotherapy field ([84,85]). One of its uses is to compare two independently calculated dose distributions for quality assurance (QA) in treatment planning. The γ index, by combining dose difference and distance-to-agreement, is able to provide the means for an efficient analysis.

For this reason, it was decided to exploit this metric along with the others mentioned above.

For each point in the dose distribution, the gamma index, γ, is calculated as:

(12) $$\gamma \left(x\right)=\min_{x^{\prime }\in \Omega }\sqrt{{\left(\frac{C\left(x\right)-C^{\prime }\left(x^{\prime }\right)}{\delta C}\right)}^{2}+{\left(\frac{x-x^{\prime }}{\delta x}\right)}^{2}}\;\;\;\;\;\;\forall x\in \Omega$$

where:

- C(x) is the true initial (or reference) dose distribution at the position x;
- $C^{\prime }\left(x^{\prime }\right)$ is the predicted initial dose distribution at the position $x^{\prime }$;
- δC is the threshold value of the dose, calculated as a percentage of the maximum value of the true initial dose distribution (global γ index [85]);
- δx is the distance threshold value in millimeters;
- Ω is the spatial domain as stated in the above sections.

A gamma index value γ<1 indicates that the reconstructed dose at the point x is within the acceptable range of the reference dose, considering both dose and spatial criteria.

As a result, the γ index calculation provides a value for each point of the dose distribution. To extrapolate a global evaluation we used the percentage of the points for which γ<1, and the average value 〈γ〉.

According to the TG-218 report [67], the tolerance limits have been set to γ passing rate ≥95%, with 3%/2 mm criteria and a 10% dose threshold.

## Figures and Tables

**Table 1:** Rectangular distribution. Error analysis with different metrics utilized as described in Section 4.4. Distances are always evaluated with respect to the true initial distribution. MSE: Mean Square Error; γ: gamma index calculated at 3%/2 mm.

Time (h)	Distribution	MSE	%γ<1	$\langle \gamma \rangle$
20	diffused	$3.44\times 10^{-4}$	100%	0.339
	predicted	$4.67\times 10^{-6}$	100%	0.0681
40	diffused	$1.13\times 10^{-3}$	82.9%	0.626
	predicted	$1.27\times 10^{-5}$	100%	0.0869
60	diffused	$2.16\times 10^{-3}$	58.7%	0.899
	predicted	$1.94\times 10^{-5}$	100%	0.0992
80	diffused	$3.35\times 10^{-3}$	44.9%	1.16
	predicted	$4.03\times 10^{-5}$	100%	0.128
100	diffused	$4.61\times 10^{-3}$	34.6%	1.41
	predicted	$8.54\times 10^{-5}$	100%	0.153

**Table 2:** Circular distribution. Error analysis with different metrics utilized as described in Section 4.4. Distances are always evaluated with respect to the true initial distribution. MSE: Mean Square Error; γ: gamma index calculated at 3%/2 mm.

Time (h)	Distribution	MSE	%γ<1	$\langle \gamma \rangle$
20	diffused	$9.24\times 10^{-4}$	100%	0.465
	predicted	$2.66\times 10^{-5}$	100%	0.118
40	diffused	$2.52\times 10^{-3}$	58.5%	0.845
	predicted	$8.87\times 10^{-5}$	100%	0.188
60	diffused	$4.28\times 10^{-3}$	42.1%	1.20
	predicted	$1.92\times 10^{-4}$	100%	0.288
80	diffused	$6.07\times 10^{-3}$	32.8%	1.53
	predicted	$4.30\times 10^{-4}$	91.4%	0.437
100	diffused	$7.83\times 10^{-3}$	25.0%	1.84
	predicted	$6.58\times 10^{-4}$	84.9%	0.538

**Table 3:** 3D distribution. Error analysis with different metrics utilized as described in Section 4.4. Distances are always evaluated with respect to the true initial distribution. MSE: Mean Square Error; γ: gamma index calculated at 3%/2 mm.

Time (h)	Distribution	MSE	%γ<1	$\langle \gamma \rangle$
40	diffused	$1.85\times 10^{-3}$	59.8%	0.905
	predicted	$9.15\times 10^{-4}$	92.4%	0.403

## Data Availability

Data will be made available on request.

## Supplementary Materials

The following supporting information can be downloaded at: https://www.mdpi.com/article/10.3390/gels10090565/s1, Figure S1: Dose-volume histograms in the case of the rectangular distributions; Table S1: Rectangular distribution. Error analysis with different discrete metrics utilized as described in Section 1 of Supplementary Materials; Table S2: Doses at 95% (D95) in the case of the rectangular distributions; Table S3: Circular distribution. Error analysis with different discrete metrics utilized as described in Section 1 of Supplementary Materials; Figure S2: Dose-volume histograms in the case of the circular distributions; Table S4: Doses at 95% (D95) in the case of the circular distributions; Table S5: 3D distribution. Error analysis with different metrics utilized as described in Section 1 of Supplementary Materials; Figure S3: Dose-volume histograms in the case of the 3D distribution. Reference [86] is cited in supplementary materials.
