# Supplementary material for: Diffusion Correction in Fricke Hydrogel Dosimeters: A Deep Learning Approach with 2D and 3D Physics-Informed Neural Network Models
Source: Gels. 2024 Aug 30;10(9):565. doi: 10.3390/gels10090565 (PMC11431587; doi:10.3390/gels10090565)
Supplement: Supplementary file 1 [file gels-10-00565-s001.zip › gels-3111508-supplementary.pdf]

Supplementary Materials: Diffusion correction in Fricke hydrogel dosimeters: a deep learning approach with 2D and 3D physics-informed neural network models

Mattia Romeo [1,2,3,‡] 0009-0002-9658-9384, Grazia Cottone [1,‡] 0000-0001-6923-6394, Maria Cristina D'Oca [1,2,4], Antonio Bartolotta [1], Salvatore Gallo [5], Roberto Miraglia [6], Roberta Gerasia [6], Giuliana Milluzzo [2], Francesco Romano [2], Cesare Gagliardo [7], Fabio Di Martino [8,9,10], Francesco d'Errico [11,12], Maurizio Marrale [1,2,4] 0000-0002-0091-3243

1. Additional metrics

1.1. Discrete metrics

The discrete metrics can be used to evaluate the overlap between two spatial distributions, but this requires a definition of ROI of the distribution. In the radiotherapy treatment plannings the ROIs are usually the organs at risk (OATs) or the planned target volumes (PTV). In our case we do not have any OAT or PTV, but we can think at our distributions as ideally optimised for treatment plans. Under this condition we can assume that the dose has maximum value within a PTV and zero value outside, and we can obtain a binary mask of a hypothetical PTV by choosing an opportune threshold value.

The type of distribution we simulated respects this assumption (i.e. it shows a bi-modal shape), allowing the identification of well-defined ROI. The optimal threshold value depends on the distribution and can depend on the user who perform the evaluation.

In this work we chose a trehshold value of 0.1. In this way we have a ROI definition which has a direct linkage with applications of the radiotherapy field.

Once we extract ROIs from the true and the predicted dose distribution, we evaluate the volume overlap using the Dice Similarity Index (DSI) and the contour matching using the Hausdorff Distances (HD). Given two volume ROIs, said A and B, the DSI is defined as:

$$DSI = 2\frac{|A \cap B|}{|A| + |B|} \tag{1}$$

where $|\cdot|$ stands for the dimension of the ROI. Said ∂A and ∂B the contours of the ROIs A e B, the HD is defined as:

$$HD = \max \left(\sup_{\mathbf{x}\in\partial A} d(\mathbf{x}, \partial B), \sup_{\mathbf{y}\in\partial B} d(\mathbf{y}, \partial A) \right) \tag{2}$$

where in general

$$d(\mathbf{x}, C) = \inf_{\mathbf{c}\in C} d(\mathbf{x}, \mathbf{c}) \tag{3}$$

with $d(\mathbf{x}, \mathbf{c})$ the Euclidean distance between $\mathbf{x}$ and $\mathbf{c}$, is the distance between a point $\mathbf{x}$ and an ensemble C.

The HD can be strongly affected by just an outlier, so it is usually accompanied by the Hausdoff distance at the 95th percentile, HD95. Let be:

$$d_k(X, Y) = K^{th}_{\mathbf{x}\in X} \left(\min_{\mathbf{y}\in Y} d(\mathbf{x}, \mathbf{y}) \right) \tag{4}$$

the k-th percentile of the order distances over $\mathbf{x} \in X$, then the HD95 between two contours ∂A and ∂B is defined as [1]:

$$HD95 = \max\left(d_{95}(\partial A, \partial B), d_{95}(\partial B, \partial A)\right). \tag{5}$$

1.2. Dosimetry metrics

When two spatial dose distributions need to be compared, for example, for quality assurance purposes, the Dose Volume Histogram (DVH) can be utilized to extract key features for comparison. In particular, if the DVH is obtained from the Planning Target Volume (PTV) Region of Interest (ROI), the doses at different percentages of volume are well-known and widely used candidates for evaluation purposes. To understand this better, consider a cumulative DVH that is scaled in percentage. In this histogram, the bin doses are plotted along the horizontal axis, while the columns represent the percentage of the volume that receives a dose greater than or equal to the corresponding bin dose. This cumulative representation allows us to determine the dose levels at various volume percentages within the PTV. We define the dose at a certain percentage as the maximum dose that percentage of the volume receives. For instance, if we are interested in the dose at 95% of the volume (D95), this would be the highest dose received by the 95th percentile of the PTV volume. This metric is crucial as it provides insight into the minimum dose that covers nearly the entire target volume, ensuring adequate coverage of the PTV. To use the dose at the percentage volume as a feature for comparison in our evaluations, we first need to extract the ROIs. This is done by following a standardized protocol to ensure consistency and accuracy in the extraction process. Once the ROIs are extracted, we can calculate and compare the relevant dose metrics. In our evaluations, we specifically use the dose at 95% of the volume (D95). This value is a critical indicator of the quality of the dose distribution, as it reflects the minimum dose received by nearly the entire PTV. By comparing the D95 values between different dose distributions, we can assess and ensure the effectiveness and consistency of the treatment plans.

2. Results

2.1. 2D PINN model predictions: rectangular distribution

In table S1 the errors of the prediction are reported with respect to the true initial condition, measured according the different discrete metrics described in Section 1. For sake of comparison, the same metrics are applied to evaluate the distance between the initial condition and the final condition obtained by evolving forward the diffusion equation over different time intervals (or *diffused*).

time (h)	Distribution	DSI	HD	HD95
20	diffused	0.974	1.000	1.000
	predicted	0.999	0.707	0.000
40	diffused	0.950	2.000	2.000
	predicted	0.998	1.000	0.707
60	diffused	0.928	3.000	3.000
	predicted	0.998	1.000	0.707
80	diffused	0.911	4.000	4.000
	predicted	0.996	1.000	0.707
100	diffused	0.901	4.000	4.000
	predicted	0.995	1.000	0.707

Table S1. Rectangular distribution. Error analysis with different discrete metrics utilized as described in Section 1. Distances are always evaluated with respect to the true initial distribution. DSI: Dice Similarity Index; HD: Hausdorff Distance; HD95 Hausdorff Distance at the 95%.

Dose-volume histograms are shown in Figure S1 in the initial profile, the profile reconstructed after 200 time steps and after 500 time steps, respectively. In Table S2 the corresponding D95 values are reported at all different final times.

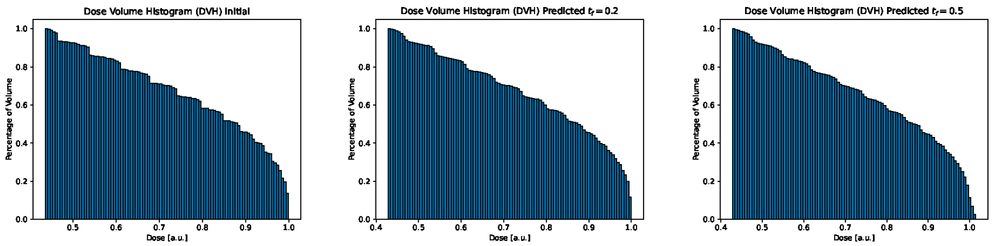

Figure S1. Dose-volume histograms in the case of the rectangular distributions. Panel a): initial distribution; panel b) backward time reconstructed initial distribution after 200 time steps ($t_f = 0.2$ a.u., i.e. 40 h) forward evolution; panel c) after 500 time steps ($t_f = 0.5$ a.u., i.e. 100 h) forward evolution.

time (h)	D95 Diffused	D95 predicted
20	0.468	0.467
40	0.437	0.468
60	0.424	0.470
80	0.428	0.470
100	0.421	0.474

Table S2. Doses at 95% (D95) in the case of the rectangular distributions. The corresponding value in the initial distribution is D95=0.464.

2.2. 2D PINN model predictions: circular distribution

The errors of the prediction with respect to the true initial condition, measured according the different metrics described in Section 1, are reported in table S3. For sake of comparison, the same metrics are applied to evaluate the distance between the initial condition and the final (*diffused*) condition obtained by evolving the diffusion equation over different time intervals.

time (h)	Distribution	DSI	HD	HD95
20	diffused	0.950	2.000	2.000
	predicted	0.994	1.000	0.707
40	diffused	0.916	3.000	3.000
	predicted	0.991	1.000	0.707
60	diffused	0.888	4.000	3.606
	predicted	0.984	1.000	1.000
80	diffused	0.863	4.472	4.472
	predicted	0.975	1.414	1.000
100	diffused	0.845	5.099	5.099
	predicted	0.965	2.000	1.414

Table S3. Circular distribution. Error analysis with different discrete metrics utilized as described in Section 1. Distances are always evaluated with respect to the true initial distribution. DSI: Dice Similarity Index; HD: Hausdorff Distance; HD95 Hausdorff Distance at the 95%.

Dose-volume histograms are shown in Figure S2 in the initial profile, the profile reconstructed after 200 time steps and after 500 time steps, respectively. In Table S4 the corresponding D95 values are reported at all times.

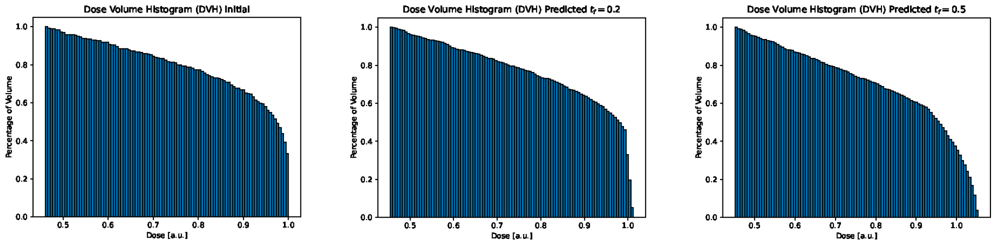

Figure S2. Dose-volume histograms in the case of the circular distributions. Panel a): initial distribution; panel b): backward time reconstructed initial distribution after 200 time steps forward evolution ($t_f = 0.2$ a.u., i.e. 40 h); panel c): after 500 time steps forward evolution ($t_f = 0.5$ a.u., i.e. 100 h)).

time (h)	D95 Diffused	D95 predicted
20	0.485	0.524
40	0.468	0.521
60	0.461	0.516
80	0.446	0.515
100	0.428	0.506

Table S4. Doses at 95% (D95) in the case of the circular distributions. The corresponding value in the initial distribution is D95=0.536.

2.3. 3D PINN model predictions: a DDP model distribution.

The errors of the prediction with respect to the true initial condition, measured according the different discrete metrics described in Section 1, are reported in table S5. As for dosimetric metrics, Dose-volume histograms are shown in Figure S3 in the initial, diffused and predicted initial dose distribution, respectively. The D95 values are 0.405 for the diffused distribution and 0.455 for the initial predicted, to be compared with D95= 0.481 in the initial condition.

time (h)	Distribution	DSI	HD	HD95
40	diffused	0.915	3.000	3.000
	predicted	0.974	3.606	1.000

Table S5. 3D distribution. Error analysis with different metrics utilized as described in Section 1. Distances are always evaluated with respect to the true initial distribution. DSI: Dice Similarity Index; HD: Hausdorff Distance; HD95 Hausdorff Distance at the 95%.

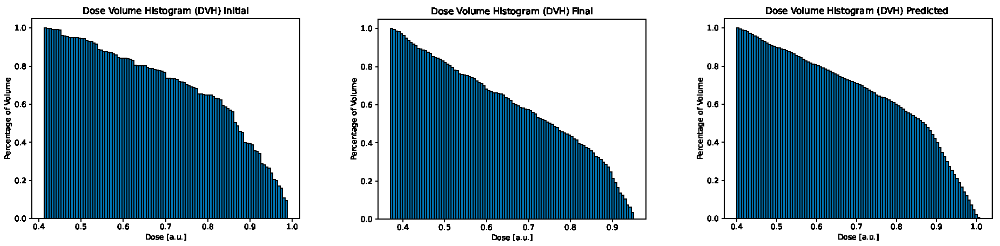

Figure S3. Dose-volume histograms in the case of the 3D distribution. Left panel: initial distribution; central panel: distribution after 200 time steps forward evolution($t_f = 0.13$ a.u., i.e. 27 h) ; right panel: backward time reconstructed initial distribution.

References

1. Huttenlocher, D.P.; Klanderman, G.A.; Rucklidge, W.J. Comparing images using the Hausdorff distance. *IEEE Transactions on pattern analysis and machine intelligence* **1993**, *15*, 850–863.

Disclaimer/Publisher's Note: The statements, opinions and data contained in all publications are solely those of the individual author(s) and contributor(s) and not of MDPI and/or the editor(s). MDPI and/or the editor(s) disclaim responsibility for any injury to people or property resulting from any ideas, methods, instructions or products referred to in the content.
